# Redevelopment of mental health first aid guidelines for supporting someone experiencing a panic attack: a Delphi study

**DOI:** 10.1186/s40359-022-00843-3

**Published:** 2022-05-27

**Authors:** Kathryn J. Chalmers, Alyssia Rossetto, Nicola J. Reavley, Anthony F. Jorm, Betty A. Kitchener, Claire M. Kelly, Amy J. Morgan, Kathy S. Bond, Fairlie A. Cottrill

**Affiliations:** 1 Mental Health First Aid Australia, Parkville, VIC Australia; 2 Centre for Mental Health, Melbourne School of Population and Global Health, University of Melbourne, Parkville, VIC Australia; 3 Kitchener Consulting, Melbourne, Australia

**Keywords:** Mental health first aid, Panic attack, Panic disorder, Prevention, Helping behaviour, Mental illness, Mental health, Mental health crisis, Delphi method, Expert consensus, Community guidelines

## Abstract

**Background:**

Panic attacks and panic disorder can have a major impact on the mental health and wellbeing of those who experience them. People with recurrent panic attacks have increased odds of developing a mental disorder and of worsening the course of existing mental disorders. Early intervention efforts at the time that a panic attack occurs might reduce or prevent some of these associated negative outcomes. Expert consensus guidelines for high income Western countries on how to provide mental health first aid for panic attacks were published in 2009. The present study aims to redevelop these guidelines to ensure content reflects current evidence and best practice.

**Methods:**

The Delphi consensus method was used to determine which helping strategies should be included in the redeveloped guidelines. A survey with items on how to assist someone who is having a panic attack was developed using the 2009 guidelines and a systematic search of grey and academic literature. Panellists with lived experience and professional experience rated these items to determine which helping statements should be included in the guidelines.

**Results:**

Thirty panellists completed all three surveys. Panellists rated 158 statements, with 83 statements meeting the criteria for inclusion in the redeveloped guidelines. The endorsed statements covered: what the first aider should know about panic attacks, what they should do if they think someone is having a panic attack, what they should do if they are uncertain whether the person is having a panic attack, what they should say and do if they know the person is having a panic attack and what they should do when the panic attack has ended.

**Conclusion:**

This study has resulted in a more comprehensive set of guidelines than the original version, with the endorsement of 83 helping actions, compared to 27 previously. The redeveloped guidelines provide greater detail on recognising the signs of a panic attack, providing initial assistance, communicating with someone experiencing a panic attack and supporting them to seek appropriate professional help if it is needed. ﻿The guidelines will be used in future updates of Mental Health First Aid training courses.

**Supplementary Information:**

The online version contains supplementary material available at 10.1186/s40359-022-00843-3.

## Background

Mental disorders are increasingly identified as leading causes of disease burden. In 2019 they accounted for 4.9% of all disability-adjusted life years (DALYs) and ranked 7th in terms of DALYs globally [1]. In the same year, anxiety disorders accounted for 22.9% of mental disorder DALYs and were a leading cause of burden worldwide, ranking as the 24th leading cause of DALYs [1]. Anxiety disorders are one of the most common categories of mental illnesses worldwide, with age-standardised prevalence varying between 2.5 to 7% by country [2] and lifetime prevalence rates as high as 33.7% [3].

Cross-national epidemiological data from 25 countries found the lifetime prevalence for panic disorder is 1.7% [4]. Panic attacks were more common, with a lifetime prevalence of 13.2%. People who experience recurrent panic attacks have increased odds of subsequently developing any mental disorder and of worsening the course of existing mental disorders [4]. Panic disorder is associated with a high degree of impairment, high levels of comorbidity with other mental disorders and other medical illnesses, and with suicidal ideation, suicidal behaviour, and completed suicide [5–8]. These findings on the increased burden of disease associated with recurrent panic attacks and panic disorder, compared to a single panic attack, suggest that early intervention efforts might reduce or prevent impairment associated with panic disorder.

It is well documented that professional mental health treatment is only received by a minority of those needing it [9–11]. A study examining the treatment gap for anxiety disorders using the results of World Mental Health Surveys found that, across 21 countries, only about a quarter (27.6%) of individuals diagnosed with an anxiety disorder received any treatment in the previous year [12].

A timely response to someone experiencing a panic attack may increase the chance of a person receiving appropriate professional assistance and it may also decrease the likelihood of them developing a mental health problem or experiencing a worsening of an existing mental health problem. Members of the public, family or friends can play a role in encouraging help seeking and engaging with professional and self help, but may lack the knowledge and skills required to do this effectively. Research has found that significant improvements are needed in the public’s knowledge and skills required to help someone experiencing a mental health problem or crisis [13–16]. In Australia, the public’s knowledge about anxiety is lower than for other mental health conditions like depression [17]. If members of the community are equipped with the appropriate skills, knowledge and confidence to recognise that someone is experiencing a panic attack, provide assistance and encourage help-seeking behaviours, they are well placed to provide early support to someone during the crisis.

The Mental Health First Aid (MHFA) program was developed in Australia in 2000 and has since spread to 24 countries [18]. It is designed to teach members of the public first aid skills. Mental health first aid is defined as “the help offered to a person who is developing a mental health problem, experiencing a worsening of an existing mental health problem or in a mental health crisis. The first aid is given until appropriate professional help is received or until the crisis resolves” [19]. A systematic review of randomised controlled trials of MHFA training courses supported the effectiveness of MHFA training in improving mental health literacy and in the amount of help offered to those with mental health problems up to 6 months after training [20]. The review found training led to improvements in mental health first aid knowledge, recognition of mental health disorders, beliefs about effective treatments and confidence in helping a person with a mental health problem.

﻿Expert consensus guidelines inform the content of the Australian MHFA course, and MHFA courses in other countries that are based on it [21]. The guidelines provide recommendations for members of the public on how to assist a person with mental health problems (e.g. depression [22]), or experiencing a mental health crisis (e.g. non-suicidal self-injury [23]). The guidelines were developed using the Delphi method, an expert consensus method that involves panel members making private, independent ratings of agreement with a series of statements [24]. The method is valuable because it can be used to incorporate practice-based evidence from experts globally and it is a feasible and ethical approach when experimental study designs cannot be used. It has been used to develop a range of mental health first aid guidelines, including the original panic attack guidelines [25]. These guidelines are available online for the public to access from the MHFA website (https://mhfa.com.au). Studies evaluating these online guidelines have found that some users found them useful in improving the quality of the support they provided to someone with a mental health problem [26, 27].

Guidelines for assisting a person experiencing a panic attack were developed in 2009 [25]. The current study aims to redevelop these guidelines to ensure that the content reflects current evidence and best practice. Guideline updates like this incorporate the latest research findings and recommendations from experts in the area of panic attacks. Recently, mental health first aid guidelines for traumatic events, depression and psychosis were redeveloped using the Delphi method [22, 28, 29] and the current redevelopment of the panic attack guidelines followed the protocol of these studies. Redeveloped mental health first aid guidelines have greater detail and recommend more first aid actions than the original guidelines, indicating that revision of the original guidelines is advisable.

This study aimed to use the Delphi method to redevelop guidelines for assisting someone experiencing a panic attack.

## Methods

﻿This study included five stages: systematic literature search, questionnaire development, panel formation, Delphi consensus survey rounds and guideline development.

### Systematic literature search

A systematic search was conducted in September 2019 by one researcher (EY, a psychology honours student), who was supervised by two other researchers (KSB, FAC) to find statements about how a member of the public can help someone experiencing a panic attack, including how to recognise the signs of a panic attack, what to do if someone is having a panic attack, how to communicate with someone experiencing a panic attack and offer short-term assistance for its duration, and how to assist them to seek appropriate professional help if it is needed. Research publications, books and online content were included in the search for relevant statements. All searches were set to return results published since 2007, as the aim was to find content that had not been covered by the literature search in the original study. We used a combination of the original search terms from 2007 and additional terms that have become more common in the intervening timeframe, as described below.

The search engines used were: Google.com, Google.com.au, Google.co.uk, Google.nz, and Google.ca. Two searches were run in each engine: (1) ‘how to help someone having attack panic attacks AND first aid OR intervention’, (2) ‘what to do if someone has a panic attack OR panic disorder’. These terms were chosen because they delivered more relevant results than other combinations. The original 2009 study used 3 searches: (1) 'panic attack' and 'self help', (2) 'panic attack' and 'first aid', and (3) 'panic attack' and (care or carer or caring).

Searches were conducted in private or incognito mode to minimise the influence of Google’s search algorithms. The search settings were adjusted each time to reflect the country of the search engine. As with previous similar Delphi studies [30], websites returned in the top 50 results from each search were reviewed as it has been found that the quality of the resources declines after the first 50 results [31]. Any further sources linked on these websites that were thought to contain useful information were also screened. Overall, 523 websites were identified for potential first aid helping actions, duplicate sites were deleted, and relevant statements were found on 22 of these sites.

The same search terms used for the Google search engines were also used to run searches within Google Scholar (see above). In addition, PsycInfo and PubMed were each used to run two searches: (1) ‘help OR support AND panic attack’, (2) ‘panic attacks AND first aid OR intervention’. In the 2009 study, the search terms 'panic attack AND intervention OR (first aid)’ were used. Searches on these databases returned a total of 299 results. Any duplicates were deleted and the remaining articles were then screened for relevance. The irrelevant articles were excluded through a hierarchical screening process, starting with titles, abstracts and then a full-text review. Following this process, 2 articles were deemed potentially relevant. Neither of these two articles read in full contained relevant statements. This was not wholly unexpected given the few results returned from database searches in previous Delphi studies that aimed to redevelop MHFA guidelines [22, 23] and further demonstrates that there is little evidence-based guidance available for members of the public wishing to support a person experiencing a panic attack.

**Fig. 1:**
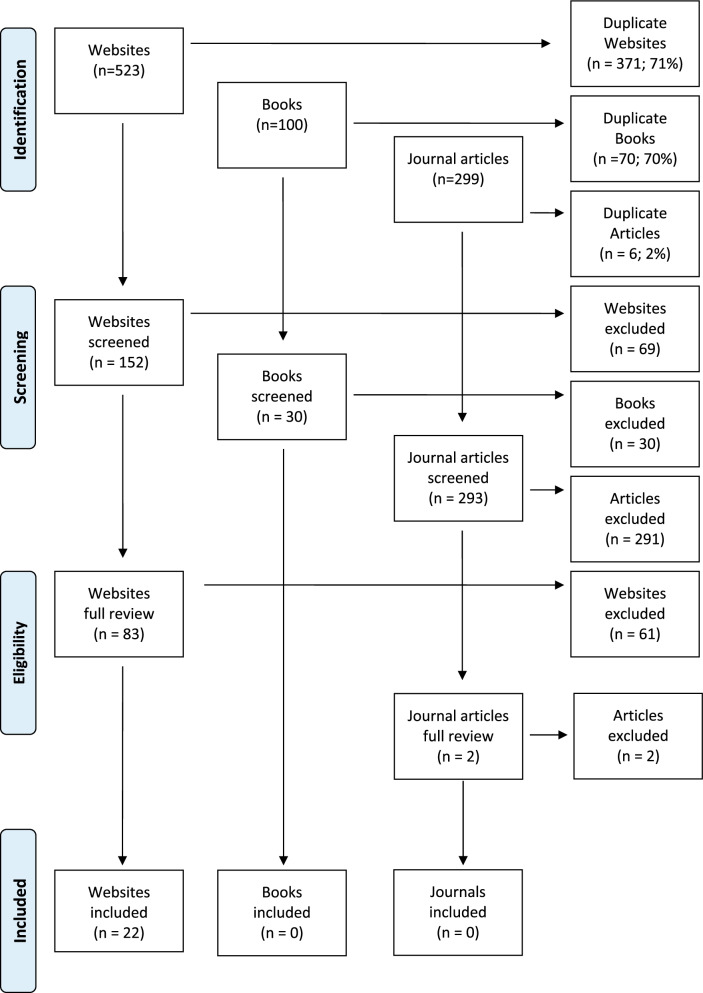
Summary of literature search

To locate relevant books, an advanced search of Google Books was conducted using the same search terms that were used on Google web search engines (see above). These two searches produced the most relevant results when tested. This differed from the 2009 study used that used Amazon to run a title and keyword search with the single term ‘panic’ [25]. The two searches returned 100 books, and 30 were screened. There were no new, relevant statements found in these books. See Fig. 1 for a summary of the literature search.

### Questionnaire development

Strategies obtained from the systematic search on providing mental health first aid to someone experiencing a panic attack were drafted into single-idea, action-oriented statements that maintained the original source’s meaning (KSB, FAC). The first questionnaire was formed using these statements, as well as some statements from the previous 2009 Delphi questionnaire. The statements included from the previous Delphi were those that were endorsed for the original guidelines, as well as those that were endorsed by 50% or more of both the original panels. All statements were sorted into thematic categories.

A working group of researchers (KJC, AR, NJR, AFJ, BAK, CMK, AJM) reviewed all collated statements. Statements were considered acceptable for inclusion in the questionnaire if the researchers agreed that they described how a first aider could assist someone experiencing a panic attack (n = 125). The working group edited the statements to improve clarity, relevance and actionability of items, for example by re-wording statements and adding examples. They did not make judgements about the content of the statements. Examples of the types of statements included in the questionnaire include:

“The first aider should give the person some space, so that they do not feel crowded.”

“If the first aider knows that the person has had a panic attack in the past, they should ask them what has worked.”

The statements that were included from the 2009 Delphi questionnaires were reviewed in consideration of the updated definition of mental health first aid [21] and the first aider’s role, as well as for comprehensibility. Some of these items were reworded to make them clearer or to capture new actions suggested by the literature.

A final review was undertaken to eliminate any remaining repetition, maximise comprehensibility and finalise the survey. Please see Additional file 1: Round 1 survey, which contains 125 items reviewed and agreed upon by the working group.

### Panel formation

Participants were recruited by KJC from high-income, western countries with developed health systems (including Australia, Canada, countries throughout Europe, Ireland, New Zealand, United Kingdom and the United States of America). Panellists were required to speak English and have professional experience in the area of panic attacks and panic disorder (e.g. as a researcher, clinician, mental health worker), or lived experience with panic attacks.

In line with the 2009 study that developed the original guidelines, the present study aimed to recruit two panels: a professional panel and a lived experience panel made up of consumers and carers. Although early Delphi studies recruited separate panels of consumers and carers, recent Delphi studies have encountered difficulty recruiting carers [22, 29]. It was therefore decided that one lived experience panel would be recruited from the outset.

A Delphi panel of at least 20 individuals has been found to yield stable results [32]. To allow for attrition over the survey rounds, this study aimed to recruit at least 30 participants to both panels. Due to challenges in recruitment these targets were not met and a decision was made by researchers to combine the lived experience group and the professional group into one panel.

Professional experts were recruited through direct editorial boards of relevant academic journals, professional bodies, mental health advocacy organisations and direct invitation, e.g. researchers and authors identified through their published work in the field. Lived experience experts were recruited through mental health advocacy organisations or direct invitation, e.g. lived experience advocates identified through their public speaking roles or authorship of websites or books. ﻿The study was also advertised through MHFA Australia’s network of Instructors, newsletter, website and social media. Experts were also asked to nominate anyone else they knew who they felt would be appropriate panel members.

﻿All individuals who expressed interest were provided with a Plain Language Statement. Confirmed panellists were invited to the Round 1 survey via email. Participants were not reimbursed for their participation.

### Delphi consensus survey rounds

The methodology used in the Delphi survey rounds, and detailed below, drew on procedures used in previous studies [22, 24, 28, 29]. Panel members completed online questionnaires, rating each statement according to how important it was that the item be included in the guidelines. A 5-point Likert scale was used (‘essential’, ‘important’, ‘don’t know/depends’, ‘unimportant’ or ‘should not be included’). Statements that achieved substantial consensus as being ‘essential’ or ‘important’ amongst the panellists were included as recommended actions for assisting someone experiencing a panic attack. Questionnaires were presented to panellists via a survey website, Survey Monkey, in three sequential rounds (see Additional files 1, 2 and 3).

﻿The data were analysed to measure the level of consensus, with statements categorised based on the following predetermined criteria:

1. Endorsed: statements rated as ‘essential’ or ‘important’ by 80% or more of the panel.
2. Re-rate: statements rated as ‘essential’ or ‘important’ by 70–79.9% of the panel.
3. Rejected: all other statements were excluded.

The Round 1 survey questionnaire collected demographic information and required participants to nominate their primary expertise in the area of panic attacks, as well as any additional expertise. In Round 1, panel members were also invited to provide open-ended feedback after each section of the questionnaire. This gave panellists the opportunity to suggest helping actions that were not already included. A working group of researchers reviewed this feedback for suggestions that contained original ideas. These suggestions were used to develop new helping statements to be included in the subsequent Round 2 questionnaire. Statements that attracted feedback suggesting ambiguity in the interpretation of their meaning were re-phrased to make them clearer. New statements were included in Round 2 alongside statements from Round 1 that met the criteria to be re-rated. ﻿In the Round 3 survey, participants re-rated any statements that were neither endorsed nor rejected in the previous round. Items not endorsed by consensus after being re-rated in Round 3 were rejected from inclusion in the guidelines.

After each round, panellists were sent individualised reports containing a summary of the results (KJC). All reports listed statements that had been endorsed for inclusion in the guidelines and statements that had been rejected. The Round 1 and 2 reports included a section presenting statements to be re-rated in the next survey round. These reports were tailored to include the individual panellist’s rating for each re-rate statement, alongside a summary rating of the combined panel for the statement to allow direct comparison of the panel member’s response to that of the group. Panel members could then decide whether to maintain or modify their ratings in the next survey round.

### Guideline development

The statements endorsed by panellists across the three survey rounds were compiled into thematic sections to form the draft guidelines (KJC). Statements were re-written as integrated text and any repetition was deleted. A working group met to finalise structure and wording (KJC, AR, NJR, AFJ, BAK, CMK, AJM). The draft guidelines were then disseminated to panellists for any final feedback and endorsement (KJC). At this point panellists could not suggest new content; however, they were invited to make suggestions that would improve clarity and reduce ambiguity.

#### Ethical considerations

The University of Melbourne Human Research Ethics Committee approved this research. Participants provided informed consent by clicking ‘yes’ to a question in the Round 1 survey.

## Results

### Participants

Thirty-four people completed Round 1. Of these 34 participants, 26 identified as female, 7 identified as male and 1 identified with another term. The average age of participants was 45.3 years (SD = 14.3, range 20–72). Participants were from Australia, Canada, France, New Zealand, Switzerland, the United Kingdom and the United States of America (see Table 1 for further demographics).

**Table 1:** Participant characteristics

	Age range (years)	Median age (years)	# Female	# Male	# Identifies with another term	# Australia	# New Zealand	# United Kingdom and Europe	# North America
Professionals (n = 15)	31–64	50.5	10	5	0	6	1	3	5
Lived experience experts (n = 19)	20–72	45.0	16	2	1	15	1	3	0
Total (n = 34)	20–72	50.0	26	7	1	21	2	6	5

Due to the small number of participants recruited for Round 1, all participants were combined into one panel. This was considered reasonable given the lived experience group and professional groups’ Round 1 responses were highly correlated. Pearson’s r was calculated to determine the correlations of endorsement rates across items between the professional and lived experience groups’ ratings. For the 125 items rated in Round 1, the item endorsement rates of the lived experience group and the professional groups were strongly correlated, r = 0.80. Previous similar Delphi studies have also used one panel, whether beginning with one diverse panel from the outset [33] or combining the panels using the strategy described above [34].

Of the 34 panellists in Round 1, 30 completed all three rounds. The participation of panellists across the three Delphi survey rounds is shown in Table 2. There were 15 panellists recruited as professionals, with the majority having multiple roles, including 4 psychiatrists, 8 psychologists, 7 academic researchers and authors, 3 mental health nurses and educators, and 1 first responder. Of 19 panellists recruited as lived experience experts, 16 selected personal lived experience as their primary expertise and 3 selected carer roles. Approximately half of all participants (56%) had another source of expertise in addition to their identified expertise, e.g. personal lived experience or carer experience as well as professional experience.

**Table 2:** Participation of Delphi panellists in each round

	Round 1	Round 2	Round 3	Retention rate (over 3 rounds)
Professionals	15	15	13	86.7%
Lived experience	19	17	17	89.5%

### Item rating

**Fig. 2:**
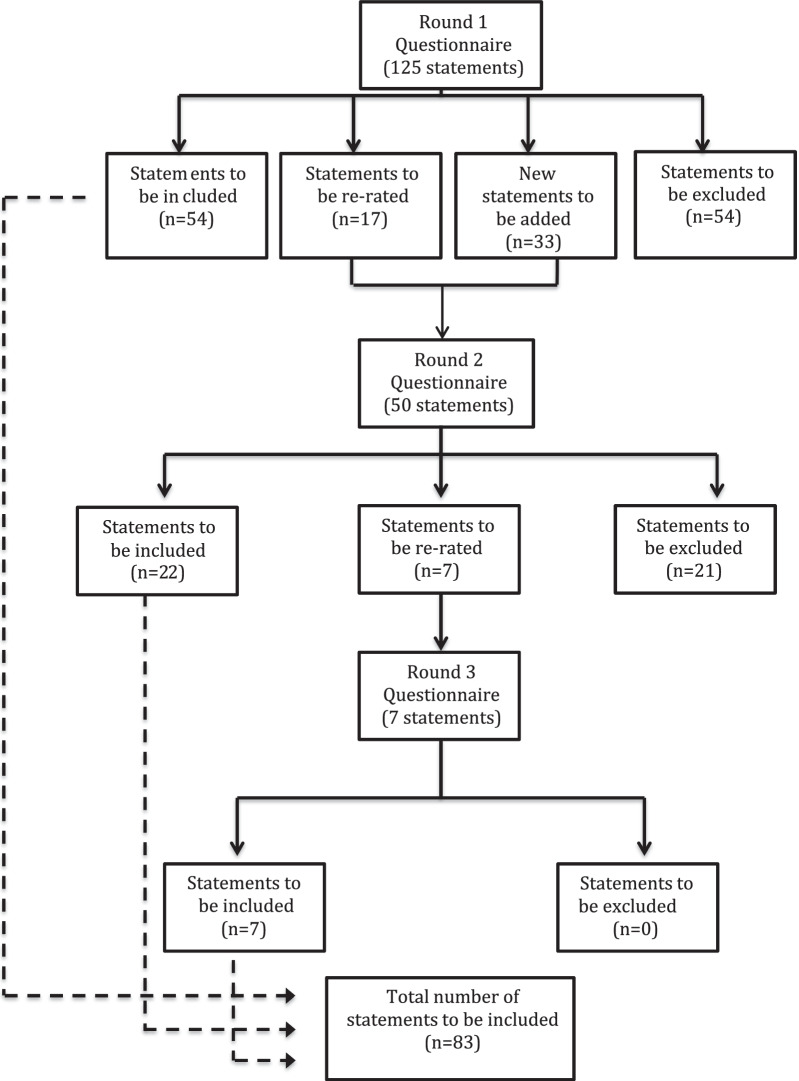
Summary of statement ratings

A total of 158 items were rated over 3 rounds with 83 items endorsed and 75 items rejected. Table 3 presents the section headings of the Delphi questionnaire and the number of items that were endorsed and rejected in each section. All statements can be viewed in Additional file 4. The rates of inclusion, exclusion and re-rating for each round are shown in Fig. 2. The endorsed items formed the basis of the guideline document entitled *Panic Attacks: Mental Health First Aid Guidelines (revised 2021)*, which is available from the Mental Health First Aid Australia website (https://mhfa.com.au).

**Table 3:** Sections in the Delphi questionnaire and number of items endorsed and rejected

Section	Topic	Number of items endorsed	Number of items rejected
1	What should the first aider know about panic attacks?	13	0
2	What should the first aider do if they think someone is having a panic attack?	5	0
3	What if the first aider is uncertain whether the person is really having a panic attack?	7	4
4	What should the first aider say and do if they know the person is having a panic attack?	50	56
5	What should the first aider say and do when the panic attack has ended?	8	15
	Total	83	75

### Difference between the 2009 and 2021 guidelines

A total of 27 items were endorsed for the 2009 guidelines compared to the 83 items endorsed for the current guidelines. There were 15 items endorsed in both the 2009 and 2021 guidelines. Five items that were endorsed in the original guidelines were not endorsed for the current guidelines, whereas six items that were not endorsed in the earlier guidelines were endorsed for the current guidelines. Some items from 2009 had adjustments to wording, including three items not endorsed in 2009 that were later endorsed in 2021. See Additional file 4 for a comparison of item ratings between the two studies.

## Discussion

This study aimed to redevelop guidelines published in 2009 for providing assistance to someone experiencing a panic attack. The expert panel reached consensus on a range of mental health first aid actions for inclusion in the guidelines, from how to support someone who is having a panic attack to when to encourage professional help.

### Comparison with original guidelines

There are some notable similarities and differences between the original guidelines and the redeveloped guidelines. The guidelines produced by the current study were formed from 83 endorsed statements, a large increase from the 27 endorsed in the 2009 study. Of the statements included from the original study, 15 were re-endorsed and 5 were not.

The general content of the redeveloped guidelines is similar to the original guidelines with both covering the knowledge required of a first aider, how to recognise a panic attack, guidance on how to provide assistance and reassurance during a panic attack, communicating with the person, and when to provide information on professional and other supports. Guidance on specific strategies during a panic attack (e.g. breathing into a paper bag, repeating coping statements, grounding techniques) were not endorsed by panellists, and perhaps not considered within the remit of a first aider, in either study. Panellists endorsed and suggested items on the importance of tailoring to the situation and person for both the original and redeveloped guidelines, e.g. asking the person how they can assist them and offering information on where to seek help if the person doesn’t know.

Similar to the original study [25], items suggesting blanket encouragement of professional help to any person who had experienced a panic attack were not highly endorsed. In Round 1, almost all items regarding what the first aider should do when the panic attack has ended were rejected from inclusion in the guidelines. In later rounds, more nuanced items that reflected panellists’ Round 1 ratings and concerns were endorsed by the combined panel, e.g. “The first aider should ask the person if they know where they can seek help and advice about panic attacks. If the person doesn’t know, the first aider should offer some suggestions”, and, “If the person says they have had recurring panic attacks, or they have changed their life to prevent panic attacks occurring, the first aider should encourage the person to see their GP or family doctor or an appropriate health professional.”

The main difference between the two sets of guidelines is that the redeveloped guidelines are more detailed, with greater consideration for the full spectrum of previous experiences of a person receiving first aid for panic attacks and the ways a first aider can tailor their responses to the person’s individual circumstances. Panellists emphasised that it may not be the person’s first panic attack and they may have a range of strategies in place to cope with panic attacks. For example, one panellist observed, “It really depends on how much lived experience of panic the person has and what the person feels comforted by.” More detail and increased guidance on tailoring assistance to what the person is experiencing is also provided in the redeveloped guidelines, e.g. helping statements on what to do if the person is disoriented or confused, cannot communicate verbally or does not know what they need.

In the 2009 Delphi, none of the items with guidance on breathing were endorsed for inclusion in the guidelines. This was because the professional panel did not endorse any of these items, though the consumer panel did. In the current study, the combined panel endorsed a total of 4 items with first aid actions using breathing techniques over the three rounds for guideline inclusion. It is difficult to know what, if anything, these differences reflect. One possible reason for this is that there has been a change in thinking regarding a first aider’s role in providing breathing guidance. This may be influenced by greater societal exposure to health and exercise pursuits that use breathing techniques as key components, e.g. stress management, meditation, yoga. This is perhaps supported by a large reduction in open-ended comments about ‘safety behaviours’ from 2021 panellists compared to 2009 panellists. In 2009, several professional panellists stated that a focus on controlled breathing may cause subsequent problems if these developed into 'safety behaviours' which interfered with treatment [25]. There may now be less concern about first aiders providing general guidance on breathing (e.g. general encouragement of slow and even breathing) whilst still recommending first aiders do not introduce specific breathing strategies (e.g. counting breaths), and this fits with the patterns of items endorsed. Whatever the reason, these differences between the original and redeveloped guidelines highlight the importance of updating guidance using current expert opinion to capture such changes.

Some of the items endorsed for the original guidelines that were not endorsed for the redeveloped guidelines might be perceived as placing the first aider as an authority on panic attacks, e.g. *The first aider should explain to the person that they are experiencing a panic attack.* The 2021 panellists expressed that the first aider instead needs to take into account that the person may have considerable knowledge and experience with panic attacks. Panellists placed an emphasis on the autonomy and previous experiences of the person in their open-ended comments, highlighting the need to follow the person’s lead in any assistance offered whilst keeping them safe, e.g. “The person should (be)…asked what they feel would help them at the time” and “A person-driven response is really important”. There has been a similar focus in other recent mental health first aid Delphi studies [28, 29]. Panellists reflected on the importance of providing reassurance and guidance to the person whilst allowing them to make decisions on managing the panic attack, e.g. “Let the person experiencing the panic attack lead if they can.” Ratings and comments highlighted the need for a first aider to strike a balance between providing the person with the autonomy to make decisions and taking the lead, whilst providing assistance if they are overwhelmed.

### Comparison between ratings of professional and lived experience groups

The professional and lived experience groups that made up the single panel in Round 1 rated items similarly. The two groups generally agreed on what helping actions were important to include in the guidelines and what items should be excluded. This included agreement about what is important that the first aider know about recognising a panic attack, how to approach someone who may be experiencing a panic attack and what to do and how to communicate with someone they know who is experiencing a panic attack. There was consensus from the groups on the need for a first aider to be calm, patient, respectful and reassuring, without making judgements or assumptions about what is happening for the person. As the two groups’ Round 1 responses were highly correlated, participants were combined into one panel for the remaining two rounds.

Some items received a notably different rating (± 20%) between the two expert groups and were endorsed by one group but not the other. In almost all cases these items were rejected by the lived experience group and appeared to be actions that might be perceived to be inappropriate during a panic attack if it was not the person’s first panic attack, if the person was already receiving professional help or if they already had coping strategies for panic attacks, e.g. “The first aider should explain to the person that they are experiencing a panic attack”, and “The first aider should try to distract the person from the panic attack, e.g. get the person talking about something else, encourage them to focus on their environment”. Open-ended comments reflected that certain actions could come across as patronising, unnecessary or harmful if the person had experienced multiple panic attacks previously.

The largest differences between the professional and lived experience groups’ opinions were in the level of support that the first aider should provide once the panic attack has ended, particularly these two items: “The first aider should offer the person information about panic attacks and resources on where to get help”, and “The first aider should encourage the person to see their GP or family doctor”. These items did not make the guidelines, as they were rejected by the combined panel (68%). The professional group endorsed both these statements (100%) and the lived experience group rejected them (42.1%). Open-ended comments indicated that panellists may have felt these were not able to be endorsed as a blanket guideline for all situations. Reasons given by panellists included that the person may have had a once-off panic attack and that the first aider should not assume they have a disorder or take any action that could distress or overwhelm the person after the panic attack has ended. Some panellists suggested it is more appropriate to check-in at a later time. However, only 37.8% of lived experience experts endorsed an item that would guide first aiders to check in with the person later that day or the following day if appropriate to the relationship, compared to 92.9% of professionals. One explanation for these results is that professional panellists were focused on the advantages of early intervention and connecting the person with professional help, whereas the lived experience experts were considering the potential harm of a first aider overloading the person with information or advice.

### Strengths

A key strength of this study is that it has resulted in updated guidelines that reflect current literature and are endorsed by experts. The use of the Delphi methodology to include the expertise of people with lived experience, as well as professional experience, and to collect practice-based evidence from experts globally is valuable and reflects recent work in the mental health field [28, 29, 35]. It was also important to redevelop these guidelines with a larger number of lived experience panellists than the original study, where lived experience representation was a key limitation.

### Limitations

A limitation of this study was that it was difficult to recruit enough participants to allow for separate expert panels, as has been used in some recent Delphi studies [30]. However, given more than half the participants had another source of expertise in addition to their identified expertise (e.g. personal lived experience or carer experience as well as professional experience), and the panel ratings were highly correlated in Round 1, the effects of this limitation were minimised.

These guidelines were developed for use in high-income Western countries with developed health care systems. Further consultation with experts from other countries and cultural backgrounds is recommended before using these guidelines within specific cultural sub-groups in Australia and in other contexts.

﻿The guidelines are also limited in that, although developed based on expert opinion, there has not been an evaluation of their use. An evaluation of these guidelines could be conducted to explore whether they are effective in guiding a first aider to provide assistance to someone experiencing a panic attack. Any training developed using these guidelines should also be evaluated.

## Conclusions

This study used the consensus of experts to update the 2009 mental health first aid guidelines for panic attacks with the most current and appropriate helping actions. This redevelopment has resulted in more detailed guidance on recognising the signs of a panic attack, providing initial assistance and communicating with someone experiencing a panic attack, and supporting them to seek appropriate professional help if it is needed.

The guidelines are available for download on the MHFA website and will be used to inform future revisions of the MHFA course. It is hoped these guidelines will increase the assistance available for people experiencing a panic attack and provide key information for those wishing to offer them help.

## Supplementary Information

**Additional file 1.** Round 1 Survey. Full survey participants completes in round one. Includes introduction given to participants, consent section and all survey items.

**Additional file 2.** Round 2 Survey. Full survey participants completes in round two. Includes introduction given to participants, consent section and all survey items.

**Additional file 3.** Round 3 Survey. Full survey participants completes in round three. Includes introduction given to participants, consent section and all survey items.

**Additional file 4.** Endorsed and rejected items. Tables presenting all statements that were endorsed as guidelines items and all statements that were rejected. Also presents a comparison between the 2009 study and the 2019 study in terms of statements supported and rejected.

## Data Availability

The datasets used and/or analysed during the current study are available from the corresponding author on reasonable request.
