# Supplementary material for: Redevelopment of mental health first aid guidelines for supporting someone experiencing a panic attack: a Delphi study
Source: BMC Psychol. 2022 May 27;10:136. doi: 10.1186/s40359-022-00843-3 (PMC9145494; doi:10.1186/s40359-022-00843-3)
Supplement: Supplementary file 1 — Additional file 1. Round 1 Survey. Full survey participants completes in round one. Includes introduction given to participants, consent section and all survey items. [file 40359_2022_843_MOESM1_ESM.pdf]

Updating the Mental Health First Aid guidelines for someone experiencing panic attacks

Information about this research

Purpose of this research

Researchers from Mental Health First Aid Australia and The Centre for Mental Health at the University of Melbourne are collaborating to update the mental health first aid guidelines for panic attacks. Mental Health First Aid Australia is a not-for-profit organisation focused on mental health training and research and The Centre for Mental Health is based at the Melbourne School of Population and Global Health at the University of Melbourne.

The aim of this current research project is to update the mental health first aid guidelines for how a member of the public should give assistance to an adult experiencing a panic attack. These guidelines are being developed for high income Western countries.

There are current mental health first aid guidelines for panic attacks that were developed in 2008. Given that they are more than 10 years old, the present study aims to update these guidelines. The guidelines will be available for download on the Mental Health First Aid Australia website (mhfa.com.au) and will be used to inform the Mental Health First Aid course curriculum.

How we are doing it

The guidelines will be formed on the basis of expert consensus of participants. Participants will complete online surveys to provide their opinions on a range of strategies for how to help someone experiencing a panic attack. Strategies that receive a high level of endorsement will be included in the guidelines.

The strategies which will be rated in the surveys are obtained from websites, books, fact sheets, brochures, scientific journal articles and training course materials. Some of the statements may seem contradictory or controversial. However, they are included because they reflect the wide range of people's beliefs about intervention and care.

What will you be asked to do?

You will be asked to complete three online surveys over about 4-6 months and the total estimated time commitment is approximately 2-3 hours.

What are the possible benefits?

This project will lead to better community support for people with mental health problems by providing guidance to Mental Health First Aid training programs. The Mental Health First Aid guidelines developed by this project will be relevant internationally for Western countries with developed health systems, and will be implemented in a range of training courses.

We cannot guarantee that people who participate in this project will gain benefit from participation, but we have found that many people report pride at having participated and are glad they were able to contribute to this important research.

What are the possible risks?

Some people may find that reading the statements upsets them, or may remind them of their own experiences. However, in our previous studies using this method, only a very small number of people reported feeling upset, and only for a short time. Many people in our previous studies have said they felt proud and happy to be able to contribute to a better understanding of how to help others. We advise anyone who finds themselves feeling upset to talk to their support people and decide whether they wish to continue with the questionnaire or stop. In the event that you feel distress and do not know where to go for help, you should contact crisis support:

Australia: Lifeline on 13 11 14

Canada: National Suicide prevention Lifeline on 1800 273 TALK (8255)

Denmark: Suicide hotline 70 201 201

Finland: SOS Crisis Centre 010 195 202

France: Suicide Écoute 01 45 39 40 00

Germany: TelephoneSeelsorge 0800/111 0 111

The Netherlands: Suicide hotline 113Online

New Zealand: Lifeline Aotearoa on 0800 543 354

Republic of Ireland: Samaritans on 116 123

Sweden: Suicide hotline 020 22 00 60

Switzerland: PARSPAS 027 321 21 21

UK: Samaritans on 08457 909090

USA: National Suicide prevention Lifeline on 1800 273 TALK (8255)

If a mental health helpline for your country is not listed here, please visit https://checkpointorg.com/global/, https://www.iasp.info/resources/Crisis_Centres/Europe/ or https://en.wikipedia.org/wiki/List_of_suicide_crisis_lines for local resources.

If you change your mind

Participation in this project is voluntary. If you change your mind about participating, you are free to withdraw from the project at any time until the last survey is closed. You may also withdraw your data if you wish, up until the individual survey is closed. Simply contact the project coordinator, Kathryn Chalmers (kathrync@mhfa.com.au).

You don't have to tell us why you have decided not to continue with the research project, but it is helpful to the researchers if you let us know that you have changed your mind about participation. This will ensure we don't keep trying to contact you.

Your privacy

We intend to protect your anonymity and the confidentiality of your responses to the fullest possible extent within the limits of the law. Any data we collect from you will be held under password protection and not given to others. We are interested in the consensus views of the panel, rather than the views of individual members. We will only present the results in statistical summary form. Upon completion of this project, the data will be de-identified. This means that all information that could identify you as the source of the data (e.g. name, email address) will be removed. Data will be kept securely for a period of 5 years, and may be destroyed after this time.

Who can I contact if I have any concerns about the project?

This research project has been approved by the Human Research Ethics Committee of The University of Melbourne. If you have any concerns or complaints about the conduct of this research project which you do not wish to discuss with the research team, you should contact the Manager, Human Research Ethics, Research Ethics and Integrity, University of Melbourne, VIC 3010. Tel: +61 3 8344 2073 or Email: HumanEthics-complaints@unimelb.edu.au. All complaints will be treated confidentially. In any correspondence, please provide the ethics ID number (**Ethics ID Number 2056861.1**) of this research project.

For more information

You received a Plain Language Statement when you expressed interest in this project (also available here). Please refer to this for more details about this study. You may also contact Kathryn Chalmers via email for further information: kathrync@mhfa.com.au.

Consent to participate

Do you meet the criteria to participate in this study?

You have been invited to participate in this research because you are 18 years or over,

AND

Have lived experience of one or more panic attacks, feel well enough to participate, AND are engaged in activities that give you a broader exposure to people's experiences of panic attacks, e.g. you are a member of a consumer advisory or advocacy group, provide peer support to others, etc.

OR

Have experience in caring for or providing day-to-day support to someone who has experienced one or more panic attacks AND be engaged in activities that give you a broader exposure to people's experiences of supporting a person who has experienced panic attacks, e.g. are a member of a carer advisory or advocacy group, provide peer support to others, etc.

OR

Are a mental health professional or researcher with at least 5 years' experience in the area of panic attacks.

* 1. Do you meet these criteria?

- ◯ Yes
- ◯ No, please exit the survey now.

Consent to participate

1. I consent to participate in this project, the details of which have been explained to me, and I have been provided with a written Plain Language Statement to keep.

2. I understand that the purpose of this research is to update the Mental Health First Aid Guidelines for Panic Attacks.

3. I understand that my participation in this project is for research purposes only.

4. I acknowledge that the possible effects of participating in this research project have been explained to my satisfaction.

5. In this project I will be required to complete three online surveys over about 4-6 months.

6. I understand that my participation is voluntary and that I am free to withdraw from this project anytime without explanation or prejudice and to withdraw any unprocessed data that I have provided.

7. I understand that the data from this research will be stored at Mental Health First Aid Australia and will be retained for a minimum of 5 years after the last publication using this data. It will not be deleted until all continued interest in the information ceases.

8. I have been informed that the confidentiality of the information I provide will be safeguarded subject to any legal requirements; my data will be password protected and accessible only by the named researchers.

9. I understand that after I consent to participating, my data will be retained by the researcher.

* 2. I understand that by submitting this survey I am giving my consent to participate in this study.

- ◯ Yes, I understand.
- ◯ I do not consent to participating in this research. Please exit the survey now.

Instructions

Definitions used in this survey

A panic attack is a distinct episode of high anxiety, with fear or discomfort, which develops abruptly and has its peak within 10 minutes.

Mental health first aid is the help offered to a person developing a mental health problem, experiencing a worsening of an existing mental health problem, or in a mental health crisis. The first aid is given until appropriate professional help is received or until the crisis resolves.

The person: the person who the mental health first aider is concerned may be experiencing a panic attack.

The first aider: a concerned family member, friend, work colleague or member of the community, who provides help to a person who may be experiencing a panic attack.

GP/Family doctor: a medical doctor based in the community who treats patients with minor or chronic illnesses and refers those with serious conditions to a specialist or hospital.

Health professional: a broad range of health professionals through which a person may seek help for panic attacks. This could include a mental health professional, GP/family doctor, or another health professional, e.g. allied health professional, hospital emergency staff.

Emergency services: services that respond to and deal with emergencies when they occur, e.g. emergency medical services (ambulance) or law enforcement (the police).

Instructions

Please complete the questionnaire by rating each statement according to how important you believe it is for inclusion in the guidelines for providing mental health first aid to someone who may be experiencing panic attacks.

Please keep in mind that the guidelines will be used by the general public. The statements need to be rated according to their importance for someone WITHOUT a counselling or clinical background.

This questionnaire should take approximately 30-60 minutes to complete. You can complete the survey in two or more sittings. Your answers are saved when you click ‘Next’ at the bottom of a page. This marks your page and you can begin again at a later date on the next page. Please be aware that once you have logged on and started responding you must complete the questionnaire on the same computer.

In the next phase of the research you will be asked to complete another two surveys over approximately 6 months. The following two surveys will be considerably shorter and take less time to complete.

Overview of the questionnaire

Section 1: What should the first aider know about panic attacks?
Section 2: What should the first aider do if they think someone is having a panic attack?
Section 3: What if the first aider is uncertain whether the person is really having a panic attack?
Section 4: What should the first aider say and do if they know the person is having a panic attack?
Section 5: What should the first aider say and do when the panic attack has ended?

Information about you

* 3. What is your name? (This allows us to determine who has completed the Round 1 survey and is therefore eligible to participate in Round 2. Your name will be deleted from your data when the project is complete).

* 4. How old are you?

* 5. What is your gender?

- Female
- Male
- I identify with another term
- Do not wish to disclose

* 6. Please indicate your primary source of expertise, i.e. lived experience, carer or professional.

- Person with a lived experience of panic attacks
- Carer or significant support person
- Professional

7. In addition to your primary source of expertise, do you also have experience with panic attacks as a:

- Person with lived experience
- Mental health carer or significant support person
- Professional

* 8. Please state the name of the organisation/s you work or volunteer for that make you eligible to participate in this study.

* 9. What is your role within the above organisation/s?

* 10. What country do you live in?

* 11. Are you a Mental Health First Aid Instructor, i.e. do you deliver the Mental Health First Aid course?

- Yes
- No

What should the first aider know about the panic attack?

This section asks you what information the first aider should know in order to assist the person whose experiencing a panic attack.

Please rate how important (from 'essential' to 'should not be included') you think it is that each statement be included in the guidelines.

Please also keep the definitions in mind when rating the statements.

What should the first aider know about panic attacks

* 12. The first aider should know the signs that indicate a person may be experiencing a panic attack.

Essential	Important	Don't know/Depends	Unimportant	Should not be included
◯	◯	◯	◯	◯

* 13. The first aider should know that symptoms of a panic attack tend to peak within several minutes.

Essential	Important	Don't know/Depends	Unimportant	Should not be included
◯	◯	◯	◯	◯

* 14. The first aider should know a panic attack usually has its peak within 10 minutes.

Essential	Important	Don't know/Depends	Unimportant	Should not be included
◯	◯	◯	◯	◯

* 15. The first aider should know that most panic attacks last between 5 and 20 minutes.

Essential	Important	Don't know/Depends	Unimportant	Should not be included
◯	◯	◯	◯	◯

* 16. The first aider should know that panic attacks often occur unexpectedly.

Essential	Important	Don't know/Depends	Unimportant	Should not be included
○	○	○	○	○

* 17. The first aider should know that panic attacks can subside as quickly as they start.

Essential	Important	Don't know/Depends	Unimportant	Should not be included
○	○	○	○	○

* 18. The first aider should know that the person can experience more than one panic attack in succession.

Essential	Important	Don't know/Depends	Unimportant	Should not be included
○	○	○	○	○

* 19. The first aider should know that a panic attack can be triggered by everyday experiences, e.g. leaving home, being in a noisy or crowded area.

Essential	Important	Don't know/Depends	Unimportant	Should not be included
○	○	○	○	○

* 20. The first aider should know that not all panic attacks have a trigger.

Essential	Important	Don't know/Depends	Unimportant	Should not be included
○	○	○	○	○

* 21. The first aider should know that the person may feel drained and exhausted after a panic attack.

Essential	Important	Don't know/Depends	Unimportant	Should not be included
○	○	○	○	○

* 22. The first aider should know that physical and emotional effects following the panic attack may last for few hours.

Essential	Important	Don't know/Depends	Unimportant	Should not be included
○	○	○	○	○

23. Do you have any comments on the above statements? Is there anything you would like to add? Please write your suggestions in the box provided.

What should the first aider do if they think someone is having a panic attack?

This section asks you what should the first aider do if they think the person is having a panic attack.

Please rate how important (from ‘essential’ to ‘should not be included’) you think it is that each statement be included in the guidelines.

Please also keep the definitions in mind when rating the statements.

When the person is having a panic attack

* 24. The first aider should know that the symptoms of a panic attack can resemble the symptoms of a heart attack or other medical condition.

Essential	Important	Don't know/Depends	Unimportant	Should not be included
○	○	○	○	○

* 25. If the first aider suspects that the person is having a panic attack, they should first ask the person if they know what is happening and whether they have had a panic attack before.

Essential	Important	Don't know/Depends	Unimportant	Should not be included
○	○	○	○	○

* 26. The first aider should identify themselves if the person does not know them.

Essential	Important	Don't know/Depends	Unimportant	Should not be included
○	○	○	○	○

27. Do you have any comments on the above statements? Is there anything you would like to add? Please write your suggestions in the box provided.

What if the first aider is uncertain whether the person is really having a panic attack?

This section asks you what if the first aider it uncertain whether the person is really having a panic attack, and not something more serious like a heart attack.

Please rate how important (from 'essential' to 'should not be included') you think it is that each statement be included in the guidelines.

Please also keep the definitions in mind when rating the statements.

What if the first aider is uncertain

* 28. If the person has not had a panic attack before, and doesn't think they are having one now, the first aider should follow physical first aid guidelines.

Essential	Important	Don't know/Depends	Unimportant	Should not be included
○	○	○	○	○

* 29. The first aider should check if the person is wearing a medical alert bracelet or pendant, or has a medical alert tattoo.

Essential	Important	Don't know/Depends	Unimportant	Should not be included
○	○	○	○	○

* 30. If the person has a medical alert, the first aider should follow the instructions on the alert.

Essential	Important	Don't know/Depends	Unimportant	Should not be included
○	○	○	○	○

* 31. The first aider should ask the person if their symptoms are stemming from anxiety or fear.

Essential	Important	Don't know/Depends	Unimportant	Should not be included
○	○	○	○	○

* 32. The first aider should ask the person if their symptoms are related to a medical problem.

Essential	Important	Don't know/Depends	Unimportant	Should not be included
○	○	○	○	○

* 33. If the person's symptoms do not subside within 15 minutes, the first aider should seek urgent medical advice.

Essential	Important	Don't know/Depends	Unimportant	Should not be included
○	○	○	○	○

* 34. The first aider should know that the person may want to seek medical attention because they are frightened by what they are experiencing.

Essential	Important	Don't know/Depends	Unimportant	Should not be included
○	○	○	○	○

* 35. The first aider should call emergency services if they are concerned that there might be a medical reason for the person's symptoms.

Essential	Important	Don't know/Depends	Unimportant	Should not be included
○	○	○	○	○

* 36. The first aider should call emergency services if the person loses consciousness or appears to be having severe difficulty breathing.

Essential	Important	Don't know/Depends	Unimportant	Should not be included
○	○	○	○	○

* 37. The first aider should know that getting emergency care may make things worse.

Essential	Important	Don't know/Depends	Unimportant	Should not be included
○	○	○	○	○

38. Do you have any comments on the above statements? Is there anything you would like to add? Please write your suggestions in the box provided.

What should the first aider say and do if they know the person is having a panic attack?

This section asks you what should the first aider say and do if they know the person is having a panic attack.

Please rate how important (from ‘essential’ to ‘should not be included’) you think it is that each statement be included in the guidelines.

Please also keep the definitions in mind when rating the statements.

Approaching the person

* 39. The first aider should tell the person that they understand what is happening to them and know how to help.

Essential	Important	Don't know/Depends	Unimportant	Should not be included
○	○	○	○	○

* 40. The first aider should gently tell the person that they may be having a panic attack.

Essential	Important	Don't know/Depends	Unimportant	Should not be included
○	○	○	○	○

* 41. The first aider should explain to the person that they are experiencing a panic attack.

Essential	Important	Don't know/Depends	Unimportant	Should not be included
○	○	○	○	○

* 42. The first aider should reassure the person that they are there to help them through what they are experiencing.

Essential	Important	Don't know/Depends	Unimportant	Should not be included
○	○	○	○	○

* 43. The first aider should ask the person directly how they can help them.

Essential	Important	Don't know/Depends	Unimportant	Should not be included
○	○	○	○	○

* 44. If the person doesn't know what they need, the first aider should reassure them that it is okay.

Essential	Important	Don't know/Depends	Unimportant	Should not be included
○	○	○	○	○

* 45. If the first aider knows that the person has had a panic attack in the past, they should ask them what has was worked.

Essential	Important	Don't know/Depends	Unimportant	Should not be included
○	○	○	○	○

* 46. The first aider should only ask questions that will guide how best to assist the person.

Essential	Important	Don't know/Depends	Unimportant	Should not be included
○	○	○	○	○

* 47. If the person refuses the first aider's help, they should ask the person if there is anyone the person would like the first aider to call for help.

Essential	Important	Don't know/Depends	Unimportant	Should not be included
○	○	○	○	○

48. Do you have any comments on the above statements? Is there anything you would like to add? Please write your suggestions in the box provided.

What should the first aider say and do if they know the person is having a panic attack? (Cont.)

This section asks you what should the first aider say and do if they know the person is having a panic attack.

Please rate how important (from ‘essential’ to ‘should not be included’) you think it is that each statement be included in the guidelines.

Please also keep the definitions in mind when rating the statements.

Approaching the person (cont.)

* 49. If the person seems unfriendly or rude, the first aider should try not to take this personally.

Essential	Important	Don't know/Depends	Unimportant	Should not be included
○	○	○	○	○

* 50. If the panic attack occurs while the person is driving, the first aider should ask them to pull over to the side of the road as soon as it is safe to do so, as they may not be able to drive safely.

Essential	Important	Don't know/Depends	Unimportant	Should not be included
○	○	○	○	○

* 51. The first aider should know that the person may not be aware of what is causing their panic attack.

Essential	Important	Don't know/Depends	Unimportant	Should not be included
○	○	○	○	○

* 52. The first aider should not pressure the person to explain what has caused them to panic.

Essential	Important	Don't know/Depends	Unimportant	Should not be included
○	○	○	○	○

* 53. The first aider should not ask the person why they are experiencing a panic attack.

Essential	Important	Don't know/Depends	Unimportant	Should not be included
○	○	○	○	○

* 54. The first aider should encourage the person to stop what they are doing and sit down.

Essential	Important	Don't know/Depends	Unimportant	Should not be included
○	○	○	○	○

* 55. The first aider should ask the person to remain still.

Essential	Important	Don't know/Depends	Unimportant	Should not be included
○	○	○	○	○

* 56. If the person wants to move around, the first aider should suggest they do some form of movement, e.g. stretch, do jumping jacks, or go for a brisk walk.

Essential	Important	Don't know/Depends	Unimportant	Should not be included
○	○	○	○	○

57. Do you have any comments on the above statements? Is there anything you would like to add? Please write your suggestions in the box provided.

What should the first aider say and do if they know the person is having a panic attack? (Cont.)

This section asks you what should the first aider say and do if they know the person is having a panic attack.

Please rate how important (from ‘essential’ to ‘should not be included’) you think it is that each statement be included in the guidelines.

Please also keep the definitions in mind when rating the statements.

Approaching the person (cont.)

* 58. If the person wants to be alone, the first aider should move away but remain visible to the person.

Essential	Important	Don't know/Depends	Unimportant	Should not be included
○	○	○	○	○

* 59. The first aider should stay with the person, unless they insist that they need to be alone.

Essential	Important	Don't know/Depends	Unimportant	Should not be included
○	○	○	○	○

* 60. The first aider should stay with the person, even if they want the first aider to leave.

Essential	Important	Don't know/Depends	Unimportant	Should not be included
○	○	○	○	○

* 61. If possible, the first aider should remain with the person until the panic attack is over.

Essential	Important	Don't know/Depends	Unimportant	Should not be included
○	○	○	○	○

* 62. The first aider should encourage the person not to run away while having a panic attack.

Essential	Important	Don't know/Depends	Unimportant	Should not be included
○	○	○	○	○

* 63. If the first aider needs to leave, they should try to find someone else to stay with the person.

Essential	Important	Don't know/Depends	Unimportant	Should not be included
○	○	○	○	○

* 64. The first aider should give the person some space, so that they do not feel crowded.

Essential	Important	Don't know/Depends	Unimportant	Should not be included
○	○	○	○	○

* 65. If there is more than one person present, the first aider should try to create a space around the person.

Essential	Important	Don't know/Depends	Unimportant	Should not be included
○	○	○	○	○

* 66. The first aider should minimise the person’s embarrassment by asking any bystanders to leave or turn away.

Essential	Important	Don't know/Depends	Unimportant	Should not be included
○	○	○	○	○

* 67. If there are people present who do not have a role in helping with the crisis, the first aider should ask them to leave.

Essential	Important	Don't know/Depends	Unimportant	Should not be included
○	○	○	○	○

68. Do you have any comments on the above statements? Is there anything you would like to add? Please write your suggestions in the box provided.

What should the first aider say and do if they know the person is having a panic attack? (Cont.)

This section asks you what should the first aider say and do if they know the person is having a panic attack.

Please rate how important (from ‘essential’ to ‘should not be included’) you think it is that each statement be included in the guidelines.

Please also keep the definitions in mind when rating the statements.

Respecting the person and their experiences

* 69. The first aider should not minimise the person's symptoms.

Essential	Important	Don't know/Depends	Unimportant	Should not be included
○	○	○	○	○

* 70. The first aider should not criticise the person for their behaviour during a panic attack.

Essential	Important	Don't know/Depends	Unimportant	Should not be included
○	○	○	○	○

* 71. The first aider should validate that what the person is experiencing is uncomfortable, terrifying and stressful.

Essential	Important	Don't know/Depends	Unimportant	Should not be included
○	○	○	○	○

* 72. The first aider should acknowledge to the person that their terror feels very real to them.

Essential	Important	Don't know/Depends	Unimportant	Should not be included
○	○	○	○	○

* 73. The first aider should not dismiss or ignore the person's panic attack.

Essential	Important	Don't know/Depends	Unimportant	Should not be included
○	○	○	○	○

* 74. The first aider should not use language that dismisses or minimises the person's experience, e.g. "Don't panic", "Don't over-react", "There is nothing to worry about", "Just calm down".

Essential	Important	Don't know/Depends	Unimportant	Should not be included
○	○	○	○	○

* 75. The first aider should let the person know that their feelings are nothing to be ashamed of.

Essential	Important	Don't know/Depends	Unimportant	Should not be included
○	○	○	○	○

76. Do you have any comments on the above statements? Is there anything you would like to add? Please write your suggestions in the box provided.

What should the first aider say and do if they know the person is having a panic attack? (Cont.)

This section asks you what should the first aider say and do if they know the person is having a panic attack.

Please rate how important (from ‘essential’ to ‘should not be included’) you think it is that each statement be included in the guidelines.

Please also keep the definitions in mind when rating the statements.

Communicating with the person

* 77. The first aider should not agree with any negative statements the person makes about themselves.

Essential	Important	Don't know/Depends	Unimportant	Should not be included
◯	◯	◯	◯	◯

* 78. The first aider should speak to the person in positive, supportive terms, e.g. "You will be okay, this will pass soon".

Essential	Important	Don't know/Depends	Unimportant	Should not be included
◯	◯	◯	◯	◯

* 79. The first aider should speak clearly and slowly.

Essential	Important	Don't know/Depends	Unimportant	Should not be included
◯	◯	◯	◯	◯

* 80. The first aider should speak to the person in a reassuring but firm manner.

Essential	Important	Don't know/Depends	Unimportant	Should not be included
◯	◯	◯	◯	◯

* 81. The first aider should use short, clear sentences.

Essential	Important	Don't know/Depends	Unimportant	Should not be included
◯	◯	◯	◯	◯

* 82. The first aider should try to be patient with the person.

Essential	Important	Don't know/Depends	Unimportant	Should not be included
◯	◯	◯	◯	◯

* 83. The first aider should try to remain calm and avoid becoming caught up in the panic.

Essential	Important	Don't know/Depends	Unimportant	Should not be included
◯	◯	◯	◯	◯

* 84. The first aider should make direct eye contact with the person.

Essential	Important	Don't know/Depends	Unimportant	Should not be included
◯	◯	◯	◯	◯

* 85. The first aider should not criticise the person for having a panic attack.

Essential	Important	Don't know/Depends	Unimportant	Should not be included
◯	◯	◯	◯	◯

* 86. The first aider should not express pity to the person.

Essential	Important	Don't know/Depends	Unimportant	Should not be included
◯	◯	◯	◯	◯

87. Do you have any comments on the above statements? Is there anything you would like to add? Please write your suggestions in the box provided.

What should the first aider say and do if they know the person is having a panic attack? (Cont.)

This section asks you what should the first aider say and do if they know the person is having a panic attack.

Please rate how important (from ‘essential’ to ‘should not be included’) you think it is that each statement be included in the guidelines.

Please also keep the definitions in mind when rating the statements.

Reassuring the person

* 88. The first aider should explain to the person that a panic attack cannot cause them to stop breathing or suffocate.

Essential	Important	Don't know/Depends	Unimportant	Should not be included
○	○	○	○	○

* 89. The first aider should reassure the person that, although the panic attack is unpleasant, it will not cause physical harm.

Essential	Important	Don't know/Depends	Unimportant	Should not be included
○	○	○	○	○

* 90. The first aider should reassure the person that a panic attack, while very frightening, is not life threatening.

Essential	Important	Don't know/Depends	Unimportant	Should not be included
○	○	○	○	○

* 91. The first aider should reassure the person that a panic attack, while very frightening, is not dangerous.

Essential	Important	Don't know/Depends	Unimportant	Should not be included
○	○	○	○	○

* 92. The first aider should tell the person that the panic attack will soon be over.

Essential	Important	Don't know/Depends	Unimportant	Should not be included
◯	◯	◯	◯	◯

* 93. The first aider should reassure the person that a panic attack usually does not last longer than ten minutes.

Essential	Important	Don't know/Depends	Unimportant	Should not be included
◯	◯	◯	◯	◯

* 94. If the person feels like they are 'going crazy', the first aider should reassure them that they are not.

Essential	Important	Don't know/Depends	Unimportant	Should not be included
◯	◯	◯	◯	◯

* 95. The first aider should encourage the person to notice and accept what their body is doing, rather than fighting against it.

Essential	Important	Don't know/Depends	Unimportant	Should not be included
◯	◯	◯	◯	◯

* 96. The first aider should let the person know that it is possible to take control.

Essential	Important	Don't know/Depends	Unimportant	Should not be included
◯	◯	◯	◯	◯

97. Do you have any comments on the above statements? Is there anything you would like to add? Please write your suggestions in the box provided.

What should the first aider say and do if they know they are having a panic attack? (Cont.)

This section asks you what should the first aider say and do if they know the person is having a panic attack.

Please rate how important (from ‘essential’ to ‘should not be included’) you think it is that each statement be included in the guidelines.

Please also keep the definitions in mind when rating the statements.

De-escalating a panic attack - Using distraction

* 98. The first aider should not try to distract the person from the panic attack.

Essential	Important	Don't know/Depends	Unimportant	Should not be included
○	○	○	○	○

* 99. The first aider should try to distract the person from the panic attack, e.g. get the person talking about something else, encourage them to focus on their environment.

Essential	Important	Don't know/Depends	Unimportant	Should not be included
○	○	○	○	○

De-escalating a panic attack - Touching

* 100. The first aider should ask the person if they would like a hug or to hold their hand.

Essential	Important	Don't know/Depends	Unimportant	Should not be included
○	○	○	○	○

* 101. The first aider should not touch the person without permission.

Essential	Important	Don't know/Depends	Unimportant	Should not be included
○	○	○	○	○

* 102. The first aider should not grab, hold, or restrain the person.

Essential	Important	Don't know/Depends	Unimportant	Should not be included
○	○	○	○	○

103. Do you have any comments on the above statements? Is there anything you would like to add? Please write your suggestions in the box provided.

What should the first aider say and do if they know they are having a panic attack? (Cont.)

This section asks you what should the first aider say and do if they know the person is having a panic attack.

Please rate how important (from ‘essential’ to ‘should not be included’) you think it is that each statement be included in the guidelines.

Please also keep the definitions in mind when rating the statements.

De-escalating a panic attack - De-escalation strategies

* 104. The first aider should fan the person to get air on their face.

Essential	Important	Don't know/Depends	Unimportant	Should not be included
○	○	○	○	○

* 105. The first aider should try to keep the person cool, e.g. use a cold object, ideally a wet washcloth on the person's neck and face.

Essential	Important	Don't know/Depends	Unimportant	Should not be included
○	○	○	○	○

* 106. The first aider should offer the person water.

Essential	Important	Don't know/Depends	Unimportant	Should not be included
○	○	○	○	○

* 107. The first aider should encourage the person to use the follow de-escalation strategies :

	Essential	Important	Don't know/ depends	Unimportant	Should not be included
*use mindfulness activities, e.g. think about their feet for a moment, notice the feel of their shoes against their skin, feel the pressure of the ground underneath them, notice any other subtle physical sensations.	○	○	○	○	○

	Essential	Important	Don't know/ depends	Unimportant	Should not be included
*focus their attention on something outside their own body and symptoms, e.g. count backwards in threes from 100, recall the words from a favourite song or concentrate on the sights and sounds around them.	○	○	○	○	○
*think of panic as a passing event similar to 'surfing a wave', it builds up, peaks and then washes up on the beach.	○	○	○	○	○
*label their fear level from zero to ten and notice that it decreases.	○	○	○	○	○
*think about a relaxing and calm environment.	○	○	○	○	○
*engage in progressive relaxation, e.g. relax each muscle from their toes all the way up to their neck and face.	○	○	○	○	○
*rub a small amount of lavender essence onto their wrist or hand and inhale.	○	○	○	○	○
*sit with their head between their knees.	○	○	○	○	○
*stamp on the spot, as this helps some people control their breathing.	○	○	○	○	○
*place their arms in the air.	○	○	○	○	○
*move to a peaceful or quiet spot.	○	○	○	○	○
*remove anything that is obviously causing distress.	○	○	○	○	○
*take small sips of water to help them calm down.	○	○	○	○	○
*repeat coping statements such as “this is just panic – it will pass”.	○	○	○	○	○

* 108. The first aider should not pressure the person to do something they do not want to do.

Essential	Important	Don't know/Depends	Unimportant	Should not be included
○	○	○	○	○

109. Do you have any comments on the above statements? Is there anything you would like to add? Please write your suggestions in the box provided.

What should the first aider say and do if they know they are having a panic attack? (Cont.)

This section asks you what should the first aider say and do if they know the person is having a panic attack.

Please rate how important (from ‘essential’ to ‘should not be included’) you think it is that each statement be included in the guidelines.

Please also keep the definitions in mind when rating the statements.

De-escalating a panic attack - Breathing

* 110. The first aider should help the person get their breathing under control.

Essential	Important	Don't know/Depends	Unimportant	Should not be included
○	○	○	○	○

* 111. The first aider should encourage the person to try to slow their breathing down gradually.

Essential	Important	Don't know/Depends	Unimportant	Should not be included
○	○	○	○	○

* 112. The first aider should encourage the person to take slow, even breaths.

Essential	Important	Don't know/Depends	Unimportant	Should not be included
○	○	○	○	○

* 113. The first aider should encourage the person to breathe in through their nose and out through their mouth.

Essential	Important	Don't know/Depends	Unimportant	Should not be included
○	○	○	○	○

* 114. The first aider should encourage the person to breathe with their whole diaphragm, not just into their lungs.

Essential	Important	Don't know/Depends	Unimportant	Should not be included
○	○	○	○	○

* 115. The first aider should suggest that the person breathe into a paper bag.

Essential	Important	Don't know/Depends	Unimportant	Should not be included
○	○	○	○	○

* 116. The first aider should not suggest that the person breathe into a paper bag.

Essential	Important	Don't know/Depends	Unimportant	Should not be included
○	○	○	○	○

* 117. If the first aider doesn't have a paper bag, they should suggest that the person cup their mouth and nose with their hand.

Essential	Important	Don't know/Depends	Unimportant	Should not be included
○	○	○	○	○

* 118. The first aider should encourage the person to close their eyes when they are using breathing strategies.

Essential	Important	Don't know/Depends	Unimportant	Should not be included
○	○	○	○	○

* 119. While the person is doing breathing exercises, the first aider should put a hand on the person's back.

Essential	Important	Don't know/Depends	Unimportant	Should not be included
○	○	○	○	○

120. Do you have any comments on the above statements? Is there anything you would like to add? Please write your suggestions in the box provided.

What should the first aider say and do if they know they are having a panic attack? (Cont.)

After de-escalating a panic attack

* 121. If the coping strategies are working for the person, the first aider should encourage the person to continue using them.

Essential	Important	Don't know/Depends	Unimportant	Should not be included
○	○	○	○	○

* 122. After the person engages in the coping strategies, the first aider should tell them that they are doing a good job.

Essential	Important	Don't know/Depends	Unimportant	Should not be included
○	○	○	○	○

123. Do you have any comments on the above statements? Is there anything you would like to add? Please write your suggestions in the box provided.

What should the first aider say and do when the panic attack has ended?

This section asks you what should the first aider say and do when the panic attack has ended.

Please also keep the definitions in mind when rating the statements.

When the panic attack has ended

* 124. After the panic attack has passed, the first aider should:

	Essential	Important	Don't know/ depends	Unimportant	Should not be included
*ask the person if they would like to talk about how they are feeling.	◯	◯	◯	◯	◯
*offer to get the person something to eat or drink.	◯	◯	◯	◯	◯
*check that the person feels okay by themselves before they leave.	◯	◯	◯	◯	◯
*remind them of how strong and brave they are.	◯	◯	◯	◯	◯
*let the person know that they can reach out to talk to them if things are getting overwhelming.	◯	◯	◯	◯	◯

* 125. After the panic attack has passed, the first aider should not ask the person about the cause.

Essential	Important	Don't know/Depends	Unimportant	Should not be included
◯	◯	◯	◯	◯

* 126. After the panic attack has passed, the first aider should check in with the person later that day or the following day, to check that they are doing okay, if appropriate to the relationship.

Essential	Important	Don't know/Depends	Unimportant	Should not be included
◯	◯	◯	◯	◯

* 127. The first aider should tell the person that effective treatments are available for panic disorder.

Essential	Important	Don't know/Depends	Unimportant	Should not be included
○	○	○	○	○

* 128. The first aider should offer the person information about panic attacks and resources on where to get help.

Essential	Important	Don't know/Depends	Unimportant	Should not be included
○	○	○	○	○

* 129. The first aider should encourage the person to learn as much as they can about panic attacks.

Essential	Important	Don't know/Depends	Unimportant	Should not be included
○	○	○	○	○

* 130. The first aider should encourage the person to see their GP or family doctor.

Essential	Important	Don't know/Depends	Unimportant	Should not be included
○	○	○	○	○

* 131. If the first aider has an ongoing relationship with the person, they should wait at least 24 hours after the panic attack before encouraging the person to see their GP or family doctor.

Essential	Important	Don't know/Depends	Unimportant	Should not be included
○	○	○	○	○

* 132. The first aider should share stories of other people they know who've worked through panic attacks with health professionals, while maintaining confidentiality.

Essential	Important	Don't know/Depends	Unimportant	Should not be included
○	○	○	○	○

133. Do you have any comments on the above statements? Is there anything you would like to add? Please write your suggestions in the box provided.

Feedback and thank you

If anything in this survey has caused you distress and you would like to talk with someone about it you can contact the appropriate crisis help line below:

Australia: Lifeline on 13 11 14

Canada: National Suicide prevention Lifeline on 1800 273 TALK (8255)

Denmark: Suicide hotline 70 201 201

Finland: SOS Crisis Centre 010 195 202

France: Suicide Écoute 01 45 39 40 00

Germany: TelephoneSeelsorge 0800/111 0 111

The Netherlands: Suicide hotline 113Online

New Zealand: Lifeline Aotearoa on 0800 543 354

Republic of Ireland: Samaritans on 116 123

Sweden: Suicide hotline 020 22 00 60

Switzerland: PARSPAS 027 321 21 21

UK: Samaritans on 08457 909090

USA: National Suicide prevention Lifeline on 1800 273 TALK (8255)

If a mental health helpline for your country is not listed here, please visit https://checkpointorg.com/global/, https://www.iasp.info/resources/Crisis_Centres/Europe/ or https://en.wikipedia.org/wiki/List_of_suicide_crisis_lines for local resources.

That is the end of the first round survey!

Thank you very much for your contribution.

By pressing the "next" button your final responses will be registered with our survey software. Once all panel members have lodged their responses, we will collate the data and send you a report on the findings and the second survey.

We are extremely grateful for your contribution.

Best Wishes,
Mental Health First Aid Australia Research Team and The Centre for Mental Health, University of Melbourne
