# Supplementary material for: Redevelopment of mental health first aid guidelines for supporting someone experiencing a panic attack: a Delphi study
Source: BMC Psychol. 2022 May 27;10:136. doi: 10.1186/s40359-022-00843-3 (PMC9145494; doi:10.1186/s40359-022-00843-3)
Supplement: Supplementary file 3 — Additional file 3. Round 3 Survey. Full survey participants completes in round three. Includes introduction given to participants, consent section and all survey items. [file 40359_2022_843_MOESM3_ESM.pdf]

Round 3 Updating the Mental Health First Aid guidelines for someone experiencing panic attacks

Introduction and Instructions

Your participation
Thank you for your participation in this project so far.

As advised, participation in this project involves completing three rounds of online surveys. Thank you for completing the first two surveys. **It is now time to complete the third and final survey. There are only 7 items to be re-rated in this survey.**

Purpose of this research
The aim of this research is to update the mental health first aid guidelines for how a member of the public should give assistance to an adult experiencing a panic attack. These guidelines are being developed for high income Western countries.

Instructions
Your task is to complete the questionnaire by rating each statement according to how important you believe it is for inclusion in the guidelines for providing mental health first aid to someone who may be experiencing panic attacks. This involves re-rating some items from Round 2. There are no new items in Round 3.

Please keep in mind that the guidelines will be used by the general public. The statements need to be rated according to their importance for someone WITHOUT a counselling or clinical background.

This questionnaire should take approximately **5 minutes** to complete. You can complete the survey in two or more sittings. Your answers are saved when you click 'Next' at the bottom of a page. This marks your page and you can begin again at a later date on the next page. Please be aware that once you have logged on and started responding you must complete the questionnaire on the same computer.

How this questionnaire was developed
The statements in this questionnaire were derived from the results of the Round 2 survey. All items in the Round 3 survey are RE-RATE items. In Round 3, re-rate items are those that were new in Round 2 and were neither endorsed or rejected. An item is rerated when 70%–79% of panel members from the expert group rated it as essential or important.

It is important to remember that we do not necessarily agree with these statements, and some may seem contradictory or controversial. The items have been included because they reflect a wide range of people's beliefs about intervention and care. Your role is to provide us with you opinion to inform the development a set of guidelines that reflect current expert opinion.

Consent to participate
It is important for you to know that participation in this study is completely voluntary. You are not under any obligation to participate and you can withdraw at any time.

We would like to thank you for your time and effort, and encourage you to provide us with feedback on this process.

Who can I contact if I have any concerns about the project?
This research project has been approved by the Human Research Ethics Committee of The University of Melbourne. If you have any concerns or complaints about the conduct of this research project which you do not wish to discuss with the research team, you should contact the Manager, Human Research Ethics, Research Ethics and Integrity, University of Melbourne, VIC 3010. Tel: +61 3 8344 2073 or Email: HumanEthics-complaints@unimelb.edu.au. All complaints will be treated confidentially. In any correspondence, please provide the ethics ID number (**Ethics ID Number 2056861.1**) of this research project.

For more information

You received a Plain Language Statement when you expressed interest in this project (also available here). Please refer to this for more details about this study. You may also contact Kathryn Chalmers via email for further information: kathrync@mhfa.com.au.

Introduction and Instructions (continued)

Definitions used in this survey

A panic attack is a distinct episode of high anxiety, with fear or discomfort, which develops abruptly and has its peak within 10 minutes.

Mental health first aid is the help offered to a person developing a mental health problem, experiencing a worsening of an existing mental health problem, or in a mental health crisis. The first aid is given until appropriate professional help is received or until the crisis resolves.

The person: the person who the mental health first aider is concerned may be experiencing a panic attack.

The first aider: a concerned family member, friend, work colleague or member of the community, who provides help to a person who may be experiencing a panic attack.

GP/Family doctor: a medical doctor based in the community who treats patients with minor or chronic illnesses and refers those with serious conditions to a specialist or hospital.

Health professional: a broad range of health professionals through which a person may seek help for panic attacks. This could include a mental health professional, GP/family doctor, or another health professional, e.g. allied health professional, hospital emergency staff.

Emergency services: services that respond to and deal with emergencies when they occur, e.g. emergency medical services (ambulance) or law enforcement (the police).

Overview of the questionnaire

Section 1: What should the first aider know about panic attacks?
Section 2: What should the first aider do if they think someone is having a panic attack?
Section 3: What if the first aider is uncertain whether the person is really having a panic attack?
Section 4: What should the first aider say and do if they know the person is having a panic attack?
Section 5: What should the first aider say and do when the panic attack has ended?

Information about you

* 1. What is your name? (This allows us to determine who has completed the survey. Your name will be deleted from your data when the project is complete).

What should the first aider say and do if they know the person is having a panic attack?

This section asks you what should the first aider say and do if they know the person is having a panic attack.

Please rate how important (from ‘essential’ to ‘should not be included’) you think it is that each statement be included in the guidelines.

Please also keep the definitions in mind when rating the statements.

Approaching the person

* 2. The first aider should NOT stop the person from what they are doing unless it puts themselves or others at risk of harm.

Essential	Important	Don't know/Depends	Unimportant	Should not be included
○	○	○	○	○

* 3. The first aider should look at the person's body language to guide them on what the person wants to do (sit still, move around) and support the person with this preference.

Essential	Important	Don't know/Depends	Unimportant	Should not be included
○	○	○	○	○

* 4. If the person declines help or wishes to manage the panic attack on their own, the first aider should respect their wishes.

Essential	Important	Don't know/Depends	Unimportant	Should not be included
○	○	○	○	○

* 5. If the first aider needs to leave, they should try to find someone else who can check on the person.

Essential	Important	Don't know/Depends	Unimportant	Should not be included
○	○	○	○	○

What should the first aider say and do when the panic attack has ended?

This section asks you what should the first aider say and do when the panic attack has ended.

Please also keep the definitions in mind when rating the statements.

When the panic attack has ended

* 6. The first aider should be aware of the range of professional help available for panic attacks.

Essential	Important	Don't know/Depends	Unimportant	Should not be included
○	○	○	○	○

* 7. The first aider should tell the person effective professional help is available for panic attacks.

Essential	Important	Don't know/Depends	Unimportant	Should not be included
○	○	○	○	○

* 8. The first aider should tell the person that if the panic attacks recur, they should see their GP or family doctor or an appropriate health professional.

Essential	Important	Don't know/Depends	Unimportant	Should not be included
○	○	○	○	○

Thank you

Thank you for sharing your expertise and time with us.

If anything in this survey has caused you distress and you would like to talk with someone about it you can contact the appropriate crisis help line below:

Australia: Lifeline on 13 11 14

Canada: National Suicide prevention Lifeline on 1800 273 TALK (8255)

Denmark: Suicide hotline 70 201 201

Finland: SOS Crisis Centre 010 195 202

France: Suicide Écoute 01 45 39 40 00

Germany: TelephoneSeelsorge 0800/111 0 111

The Netherlands: Suicide hotline 113Online

New Zealand: Lifeline Aotearoa on 0800 543 354

Republic of Ireland: Samaritans on 116 123

Sweden: Suicide hotline 020 22 00 60

Switzerland: PARSPAS 027 321 21 21

UK: Samaritans on 08457 909090

USA: National Suicide prevention Lifeline on 1800 273 TALK (8255)

If a mental health helpline for your country is not listed here, please visit https://checkpointorg.com/global/, https://www.iasp.info/resources/Crisis_Centres/Europe/ or https://en.wikipedia.org/wiki/List_of_suicide_crisis_lines for local resources.

This was the final survey.

By pressing the "next" button your final responses will be registered with our survey software.

Thank you for your participation, we are extremely grateful for your contribution.

Best Wishes,
Mental Health First Aid Australia Research Team and The Centre for Mental Health, University of Melbourne
